# A bibliometric analysis of HER2-positive breast cancer: 1987–2024

**DOI:** 10.3389/fonc.2024.1355353

**Published:** 2024-05-01

**Authors:** Sherlissa Ali-Thompson, Gordon R. Daly, Gavin P. Dowling, Conor Kilkenny, Luke Cox, Jason McGrath, Ma’en M. AlRawashdeh, Sindhuja Naidoo, Colm Power, Arnold D. K. Hill

**Affiliations:** ^1^ Department of Surgery, Royal College of Surgeons in Ireland, University of Medicine and Health Sciences (RCSI), Dublin, Ireland; ^2^ Department of Surgery, Beaumont Hospital, Dublin, Ireland

**Keywords:** HER2+, breast cancer, anti-HER2, trastuzumab, bibliometric, analysis

## Abstract

**Aim:**

The overamplification of human epidermal growth factor (HER2) in breast cancer (BC) has been the subject of numerous research publications since its discovery in 1987. This is the first bibliometric analysis (BA) conducted on HER2-positive (HER2+) BC. The purpose of this BA is to analyze the published research on HER2+ BC from 1987 to 2024, highlighting the most significant scientific literature, as well as the main contributing authors and journals, and evaluating the impact of clinical and lab-based publications on HER2+ BC research.

**Methods:**

The Web of Science Core Collection (WoSCC) was searched using the terms “Breast cancer” OR “Breast carcinoma” OR “Breast tumor” AND “HER2 positive” OR “HER2+”. The search was limited by publication year (1987–2024) and only full English articles were included. WoS returned 7,469 relevant results, and from this dataset, a bibliometric analysis was conducted using the “analyze results” and “journal citation report” functions in WoS and the VOSviewer 1.6.16 software to generate bibliographic coupling and co-citation analysis of authors.

**Results:**

The analysis encompassed a total of 7,469 publications, revealing a notable increase in the annual number of publications, particularly in recent years. The United States, China, Italy, Germany, and Spain were the top five most prolific countries. The top five significant institutions that published HER2+ research were the University of Texas System, Unicancer, UTMD Anderson Cancer Center, Harvard University, and University of California System. *Breast Cancer Research and Treatment*, *Clinical Cancer Research*, and *Clinical Breast Cancer* were the top three notable journals with the highest number of HER2+ BC publications. Dennis Slamon (Nc = 45,411, H-index = 51) and Jose Baselga (Nc = 32,592, H-index = 55) were the most prolific authors. Evolving research topics include anti-HER2 therapy in the neoadjuvant setting, treatment of metastatic HER2+ BC, and overcoming therapy resistance.

**Conclusion:**

This study provides an overview of HER2+ BC research published over the past three decades. It provides insight into the most cited papers and authors, and the core journals, and identifies new trends. These manuscripts have had the highest impact in the field and reflect the continued evolution of HER2 as a therapeutic target in BC.

## Introduction

Breast cancer (BC) has the highest incidence of all cancers worldwide and is the leading cause of cancer death in women (1). It accounts for 12.5% of new annual cancer cases and has an estimated mortality rate of 6.9% (2, 3). BC can be classified by molecular subtype based on the expression of estrogen receptor (ER), progesterone receptor (PR), and human epidermal growth factor receptor 2 (HER2) on immunohistochemistry (4). These subtypes include luminal type A (ER+, PR+, HER2−), luminal type B (ER+, PR−/high Ki67, HER2+/−), HER2 subtype (ER−, PR−/low Ki-67, HER2+), and triple-negative breast cancer (TNBC) subtype (ER−, PR−/low Ki67, HER2−) (4). In the era of personalized medicine, BC molecular subtype hugely influences overall treatment, targeted therapeutics, and prognosis.

HER2+ BC constitutes approximately 10%–15% of BCs (2). HER2 is a tyrosine kinase receptor present on breast cells for the normal proliferation of breast tissue. The overamplification of HER2 leads to increased proliferation and activation of proto-oncogenic pathways (4). In comparison to the luminal subtypes, HER2+ BC has a higher proliferation rate, higher recurrence rate, and higher tendency to metastasize, with up to 30%–50% of HER2+ BC patients developing brain metastases (4). The result is that only TNBC has a worse prognosis than HER2+ BC (4).

The advent of targeted therapies for HER2+ BC has improved the outcomes and prognosis of the disease. The initial therapy, trastuzumab (Herceptin®), is a monoclonal antibody that directly targets HER2 (5). In its seminal trial, the addition of trastuzumab to chemotherapy saw an increase in median survival from 20.3 months to 25.1 months (*p* = 0.046) (5), revolutionizing the treatment of HER2+ BC. Anti-HER2 antibodies continue to have a pivotal role in the treatment of HER2+ BC. A 2022 population-based cohort study evaluating women with T1a/bN0M0 HER2+ BC reported that there was a 5-year disease-free survival of 94.8% in women who received adjuvant trastuzumab in comparison to 82.7% in women who did not receive trastuzumab (6). There was also a 5-year overall survival of 100% in the women who received trastuzumab compared to 90.4% of women who did not receive trastuzumab. Since the development of trastuzumab, new monoclonal antibodies (i.e., pertuzumab) have been developed with advancements in the cell signaling cascades leading to improved progression-free and overall survival (7). The current general first-line regimen for HER2+ BC is a single-agent chemotherapeutic in combination with trastuzumab and pertuzumab (8). Therapies such as tyrosine kinase inhibitors (e.g., lapatinib and neratinib) are also being used in the treatment of HER2+ BC. The treatment of HER2+ BC brain metastases is varied. Treatment decisions are often individualized based on expert opinion. However, treatment usually consists of a combination of systemic anti-cancer treatments, radiotherapy, and surgery (7).

In this study, we performed a bibliometric analysis of the most significant scientific literature published on HER2+ BC from 1987 to 2024 in order to evaluate the impact and analyze the trends of both clinical and lab-based research publications on this topic. While bibliometric analyses have been performed on several topics in BC (9–11), this is the first study undertaken to determine the most influential literature in HER2+ BC. This study provides a succinct analysis and summary of the most-cited papers, authors, and core journals on HER2+ BC, aiming to provide insight into the evolution of the HER2+ BC literature, and how this progression has impacted the treatment of HER2+ BC.

## Materials and methods

The Web of Science Core Collection (WoSCC) was searched using the terms “Breast cancer” OR “Breast carcinoma” OR “Breast tumor” AND “HER2 positive” OR “HER2+”, yielding 20,049 results. The search was refined to include only English articles from publication years 1987–2024. This search gave 7,469 results. The WoSCC software was then used to categorize the results and retrieve the number of publications and H-index for authors, years, countries, and journals. The results were used to identify the current globally approved HER2+ BC treatment. The NIH clinicaltrials.gov database was used to identify the clinical trials and national clinical trial (NCT) number. Studies were exported to Microsoft Excel to chart a bar graph based on the number of publications per year. WoSCC Journal Citation Reports was used to record the 5-year Journal Impact factor and Eigenfactor Score for each journal. The VOSviewer 1.6.16 software was used to generate bibliographic coupling analyses, co-citation analysis of authors, and co-occurrence analysis of keywords.

## Results

### Annual number of publications

**Figure 1:**
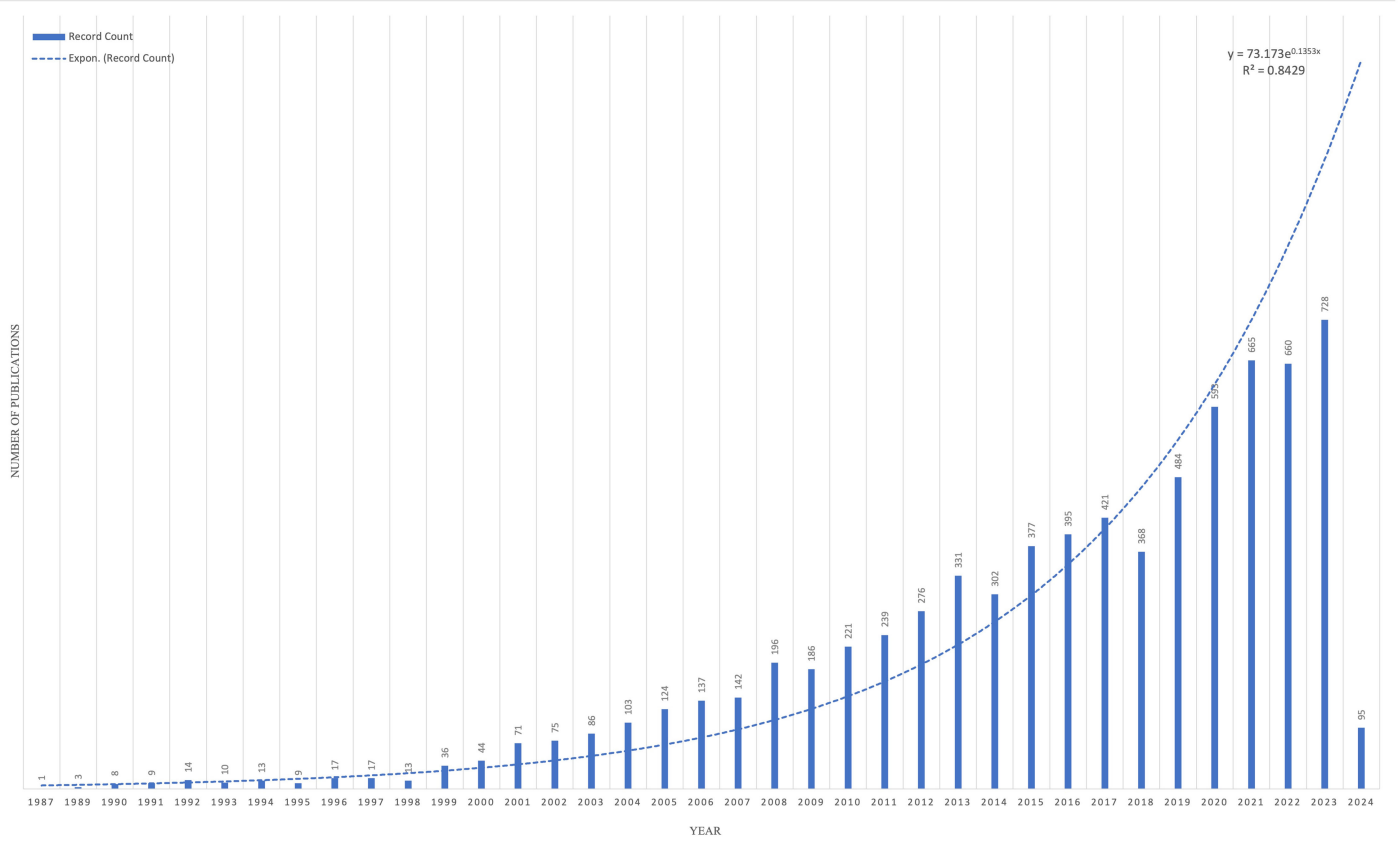
The trend in publication on HER2+ breast cancer since the discovery of HER2.

**Figure 1** represents the annual number of articles published on HER2+ BC since its discovery in 1987. In total, 7,469 articles have been published on HER2+ BC. The annual publication rate has increased exponentially (*y* = 73.173e^0.1353^ *^x^*, *R* ^2 ^= 0.9238), representing HER2’s continually evolving role in precision oncology.

### Countries

**Figure 2:**
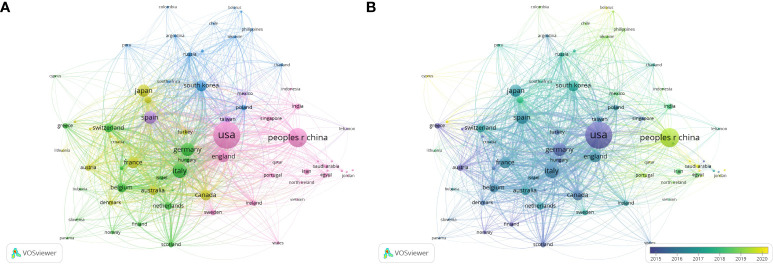
Visualization of countries involved in HER2+ BC research. **(A)** Visualization of cooperation between countries. **(B)** Visualization of temporal cooperation overlay between regions.

A total of 101 countries have contributed to publication on HER2+ BC. The top three contributors have been the USA with 2,570 publications (34%), China with 1,291 publications (17%), and Italy with 761 publications (10%) (summary in **Table 1**). However, after the USA (H-index 180, Nc 192,016), Germany had the highest H-index (107) and Nc (65,528). VOSviewer was used to visualize co-authorship between countries. Countries with at least five publications were included (75 countries). Increasing node size represents the number of articles, and the thickness of the line represents the degree of cooperation. The USA occupied the central position and shared co-authorship with England, Germany, Spain, and China, among others. The three countries with the strongest link strength were the USA (2,570 articles, 19,2016 citations, total link strength 3,229), Germany (630 articles, 65,528 citations, total link strength 1,937), and Spain (562 articles, 56,396 citations, total link strength 1,820). Despite China having the second most published articles, it ranked 14th in terms of cooperation with other nations (link strength 642) (summary in **Figure 2A**). The USA had its highest APY in 2016. Similar APY trends are observed in other Western countries. Conversely, China, along with several other Asian and Arab countries, had its highest APY in 2023 (summary in **Figure 2B**).

**Table 1:** The top 10 countries with the highest number of publications.

Rank	Countries/Regions	Count	% of 7,469	Nc	H-index
1	USA	2570	34	192,016	180
2	CHINA	1291	17	22,108	60
3	ITALY	761	10	47,795	93
4	GERMANY	630	8	65,528	107
5	SPAIN	562	7	56,396	96
6	JAPAN	528	7	21,915	61
7	FRANCE	467	6	41,782	85
8	ENGLAND	455	6	45,470	95
9	SOUTH KOREA	423	5	28,406	68
10	CANADA	397	5	49,656	82

### Journals and authors

Articles in the field of HER2+ BC were published in 1,038 journals; 120 of these journals published 10 or more articles. **Table 2** shows the journals with the highest quantity of publications and number of citations (Nc). The top three journals with the highest quantity were *Breast Cancer Research and Treatment* (481), *Clinical Cancer Research* (221), and *Clinical Breast Cancer* (168). In respect to Nc and H-index, *Clinical Cancer Research* (Nc = 15,806, H-Index = 72) held the number one position, followed by *Breast Cancer Research* (Nc = 13,248, H-Index = 59) and *Annals of Oncology* (Nc = 12,352, H-Index = 68).

**Table 2:** The top 10 journals with the highest number of publications.

Rank	Journal	Count	% of 7,469	Nc	H-index	5-year impact factor	Eigenfactor
1	*Breast Cancer Research and Treatment*	481	6.44	13,248	59	4.4	0.02445
2	*Clinical Cancer Research*	221	2.96	15,806	72	12.5	0.11111
3	*Clinical Breast Cancer*	168	2.25	2,575	27	3.3	0.00582
4	*Breast*	160	2.14	2,901	31	4.1	0.00867
5	*Annals of Oncology*	159	2.13	12,352	68	32.4	0.10839
6	*BMC Cancer*	148	1.98	3,615	32	4.3	0.0517
7	*Frontiers in Oncology*	132	1.77	681	12	5.2	0.097
8	*PLoS One*	131	1.75	2,852	28	3.8	0.71257
9	*Anticancer research*	126	1.69	1,998	24	2.2	0.0148
10	*Cancers*	123	1.65	902	16	5.6	0.12236

A total of 34,790 authors contributed to the field of HER2+ research of varying impacts. **Table 3** shows the top 10 authors with the most publications and citations. The top three authors with the highest number of articles were Jose Baselga (91 articles), Seock-Ah Im (77 articles), and Nadia Harbeck 73 articles). However, the authors with the highest Nc and H-index, which gives insight into the citation impact and quality of research, were Dennis Slamon (Nc = 45,411, H-index = 51), Jose Baselga (Nc = 32,592, H-index = 55), and Axel Ullrich (Nc = 17,068 H-index= 7).

**Table 3:** The top 10 authors with the most publications and Nc.

Rank	Authors	Record count	% of 7,469	H-index	Authors	Nc	H-index
1	J Baselga	91	1.2	55	DJ Slamon	45,411	51
2	SA Im	77	1	30	J Baselga	32,592	55
3	N Harbeck	73	0.98	32	A Ullrich	17,068	7
4	N Masuda	73	0.98	21	SG Wong	16,284	3
5	H Iwata	70	0.94	22	WJ Levin	16,203	2
6	BH Xu	68	0.91	20	S Shak	13,690	8
7	M Untch	67	0.9	41	GM Clark	12,967	9
8	SB Kim	66	0.88	25	WL Mcguire	12,216	8
9	HS Rugo	65	0.87	31	L Norton	11,591	17
10	A Prat	57	0.83	25	V Paton	10,850	3

### Co-cited references and top 10 cited papers

**Figure 3:**
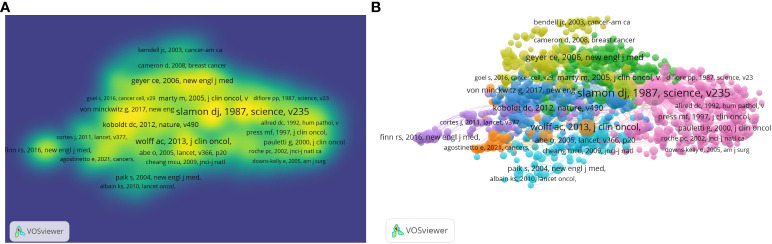
Visualization of article citations in HER2+ BC research. **(A)** Visualization of paper citation density. **(B)** Visualization of network co-citation between articles.

**Figures 3A, B** illustrate the density visualization and network visualization respectively of co-citation references in HER2 papers. The figure shows that the Slamon, DJ 1987 article has the largest cluster, indicating the highest citation co-citation count. **Table 4** shows the top 10 co-cited references in HER2+ BC. The top 3 publications with the highest citation frequencies were all by the author Slamon, DJ (1987) (*n* = 1,749), (2001) (*n* = 1,417), and (1989) (*n* = 942).

**Table 4:** The top 10 co-cited references based on citation counts.

Rank	Citation count	References	DOI
1	1,749	Slamon DJ, 1987, *Science*, v235, p177	doi 10.1126/science.3798106
2	1,417	Slamon DJ, 2001, *New Engl J Med*, v344, p783	doi 10.1056/nejm2001031534411
3	942	Slamon DJ, 1989, *Science*, v244, p707	doi 10.1126/science. 2470152
4	772	Romond EH, 2005, *New Engl J Med*, v353, p1673	doi 10.1056/nejmoa052122
5	730	Piccart-Gebhart MJ, 2005, *New Engl J Med*, v353, p1659	doi 10.1056 /nejmoa052
6	627	Wolff AC, 2013, *J Clin Oncol*, v31, p3997	doi 10.1200/jco.2013.50.9984 10.5858
7	509	Vogel CL, 2002, *J Clin Oncol*, v20, p719	doi 10.1200/jco.2002.20.3.719
8	455	Geyer CE, 2006, *New Engl J Med*, v355, p2733	doi 10.1056 /nejmoa064320
9	453	Verma S, 2012, *New Engl J Med*, v367, p1783	doi 10.1056/nejmoa1209124
10	451	Gianni I, 2012, *Lancet Oncol*, v13, p25	doi 10.1016/ 1470-2045(11)70336-9

The top 10 most cited papers on HER2+ BC are summarized in **Table 5**. The top three most cited papers were all written by Denis Slamon, representing the discovery of HER2 and its initial therapeutic implications. “Human-breast cancer—Correlation of relapse and survival with amplification of the HER-2 neu oncogene” was published in *Science* in 1987 (IF 56.9) and has been cited 9,708 times, “Use of chemotherapy plus a monoclonal antibody against HER2 for metastatic BC that overexpresses HER2” was published in the *New England Journal of Medicine* (IF 158.9) in 2001 and has been cited 8,923 times, and “Studies of the HER-2/Neu proto-oncogene in human-breast and ovarian-cancer” was published in *Science* (IF-56.9) in 1989 and has been cited 6,186 times. Summarized in **Figure 3**, Slamon’s 1987 paper holds the central position, with other seminal articles such as Von Minckwitz’s paper “Adjuvant pertuzumab and trastuzumab in early HER2-positive breast cancer”, Geyer’s paper “Lapatinib plus capecitabine for HER2-positive advanced breast cancer”, and Goel’s paper, “Overcoming therapeutic resistance in HER2-positive breast cancers with CD4/6 inhibitors”.

**Table 5:** The top 10 most cited HER2+ research articles.

Rank	Title	First author	Senior author	Institution	Country	Journal	IF (2022)	Year	No. of citations
1	Human-breast cancer—correlation of relapse and survival with amplification of the HER-2 neu oncogene	Slamon, DJ	Slamon, DJ	University of California System	USA	*Science*	56.9	1987	9708
2	Use of chemotherapy plus a monoclonal antibody against HER2 for metastatic breast cancer that overexpresses HER2	Slamon, DJ	Slamon, DJ	University of California System	USA	*New England Journal of Medicine*	158.5	2001	8293
3	Studies of the HER-2/Neu proto-oncogene in human-breast and ovarian-cancer	Slamon, DJ	Slamon, DJ	University of California System	USA	*Science*	56.9	1989	6186
4	Trastuzumab plus adjuvant chemotherapy for operable HER2-positive breast cancer	Romond, EH	Geyer, CE	University of Pittsburgh	USA	*New England Journal of Medicine*	158.5	2005	4088
5	Trastuzumab after adjuvant chemotherapy in HER2-positive breast cancer	Piccart-Gebhart	Piccart-Gebhart	Institut Jules Bordet	Belgium	*New England Journal of Medicine*	158.5	2005	3822
6	Lapatinib plus capecitabine for HER2-positive advanced breast cancer	Geyer, Charles E.	Geyer, CE	Allegheny General Hospital	USA	*New England Journal of Medicine*	158.5	2006	2502
7	Efficacy and safety of trastuzumab as a single agent in first-line treatment of HER2-overexpressing metastatic breast cancer	Vogel, CL	Vogel, CL	University of Miami	USA	*Journal of Clinical Oncology*	45.4	2002	2453
8	Trastuzumab emtansine for HER2-positive advanced breast cancer	Verma, Sunil	Verma, Sunil	University of Toronto	Canada	*New England Journal of Medicine*	158.5	2012	2327
9	Multinational study of the efficacy and safety of humanized anti-HER2 monoclonal antibody in women who have HER2-overexpressing metastatic breast cancer that has progressed after chemotherapy for metastatic disease	Cobleigh, MA	Cobleigh, MA	Rush University	USA	*Journal of Clinical Oncology*	45.4	1999	2188
10	Adjuvant trastuzumab in HER2-positive breast cancer	Slamon, DJ	Slamon, DJ	University of California System	USA	*New England Journal of Medicine*	158.5	2011	1801

### Bibliographic coupling analysis of institutions

**Figure 4:**
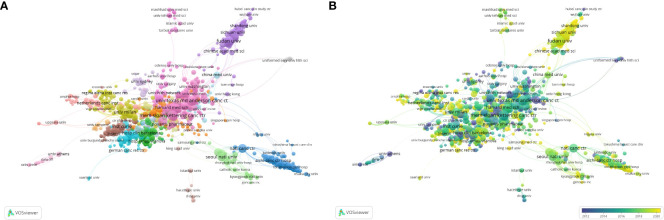
Visualization of institutions involved in HER2+ BC research. **(A)** Visualization of cooperation between institutions. **(B)** Visualization of temporal cooperation overlay between institutions.

A total of 9,577 institutions contributed to publication on HER2+ BC. The three institutions with the most publications were the University of Texas System (414), Unicancer (347), and University of Texas MD Anderson Cancer Centre (339). However, the University of California System had the highest Nc (61,133) (summary in **Table 6**). VOSviewer was used to visualize co-authorship and collaboration between institutions (**Figure 4**), including institutions with a minimum of five published articles (1,266). Memorial Sloan Kettering Cancer Center (213 articles, 29,574 citations, total link strength 994), Dana Farber Cancer Institute (151 articles, 18,227 citations, total link strength 890), and the University of Texas MD Anderson Cancer Center (204 articles, 13,888 citations, total link strength 890) had the most cooperation of any institutions in the field of HER2+ BC research. Similar to the distribution of publications by country, the institutions from the USA and Europe had their highest APYs ~2016, while Chinese institutions were most published ~5 years later.

**Table 6:** The top 10 organizations with the highest number of publications.

Rank	Affiliations	Record count	% of 7,469	Nc	H-index
1	University of Texas System	414	5.54	48,023	90
2	Unicancer	347	4.65	34,654	81
3	UTMD Anderson Cancer Center	341	4.57	32,665	80
4	Harvard University	339	4.54	38,750	88
5	University of California System	310	4.15	61,133	87
6	Roche Holding	251	2.88	27,329	27
7	Memorial Sloan Kettering Cancer Center	233	3.12	32,562	74
8	Dana Farber Cancer Institute	220	2.94	30,963	73
9	Fudan University	167	2.23	5,121	29
10	Harvard Medical School	163	2.18	13,123	55

### Co-occurrence analysis of keywords

**Figure 5:**
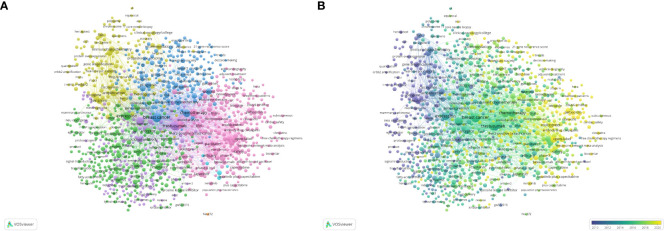
Visualization of keywords in HER2+ BC research. **(A)** Visualization of network co-citation of keywords. **(B)** Visualization of temporal co-citation overlay between keywords.

**Figure 6:**
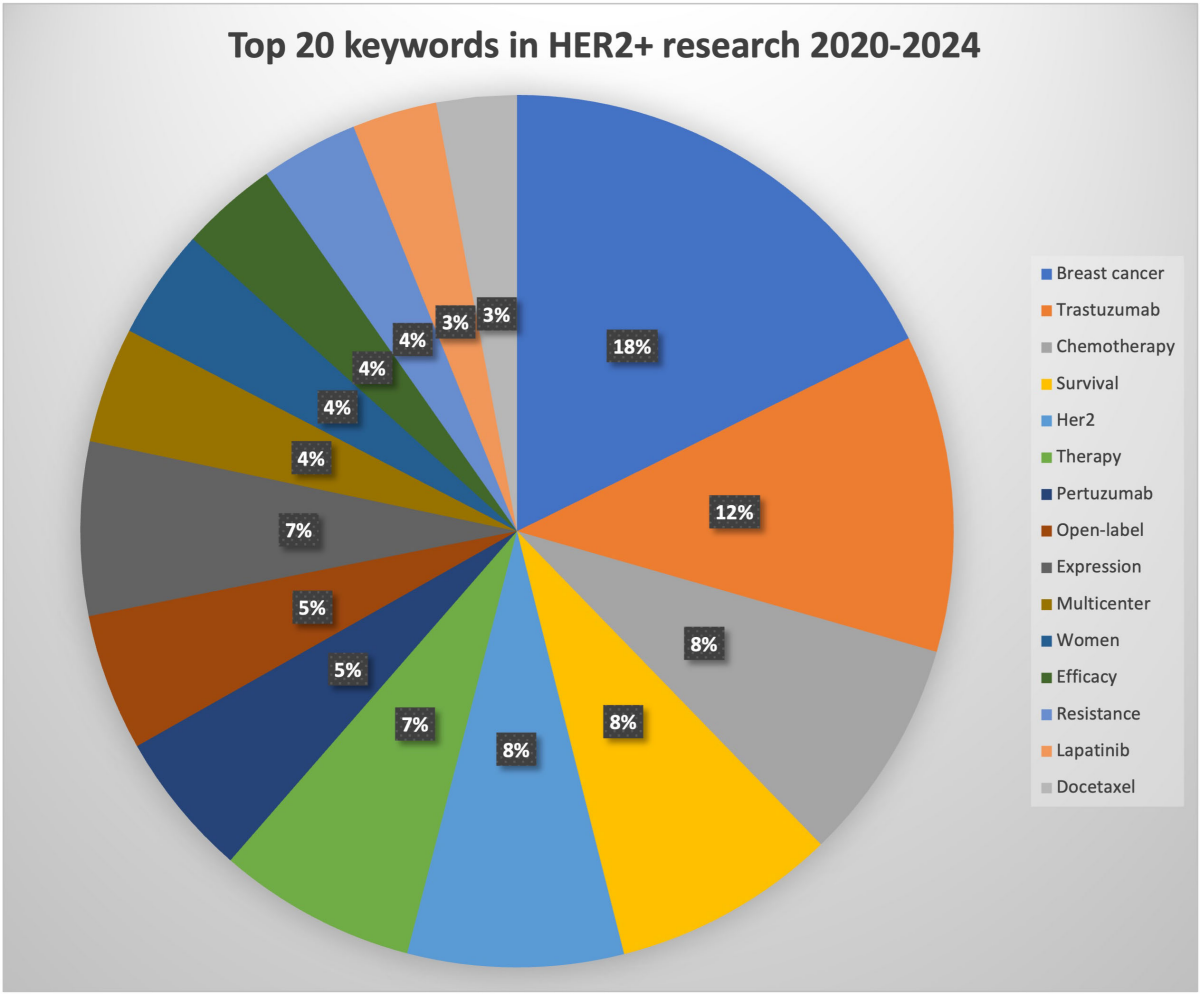
Pie chart visualization of the main topics of HER2+ articles focused on between 2020 and 2024.

The top 20 used keywords are listed in **Table 7**. Unsurprisingly, the top keywords were “breast cancer” (2,824 articles, total link strength 2815), “trastuzumab” (2,077 articles, total link strength 2072), and “HER2” (1,673 articles, total link strength 1673). Network analysis of keywords, visualized using VOSviewer in **Figure 5A**, revealed that these terms, along with chemotherapy and expression, were central hubs. **Figure 5B** reveals that newer terms of importance include antibody–drug conjugates (ADCs), lapatinib plus paclitaxel, the HERA trial, and cardiac safety. The top 15 topics focused on in the last 10 years are shown in **Table 8** and **Figure 6**. The top three discussion topics were adjuvant pertuzumab and trastuzumab, trastuzumab emtansine, and neratinib after trastuzumab-based adjuvant therapy.

**Table 7:** Top 20 keywords in HER2+ research with the strongest strength links.

Rank	Keywords	Frequency	Total link strength
1	Breast cancer	2,824	2,815
2	Trastuzumab	2,077	2,072
3	HER2	1,673	1,673
4	Therapy	1,370	1,366
5	Expression	1,364	1,364
6	Chemotherapy	1,333	1,332
7	Survival	1,196	1,195
8	Amplification	642	642
9	Adjuvant chemotherapy	611	611
10	Efficacy	577	577
11	Receptor	571	570
12	Women	571	565
13	Resistance	565	542
14	Monoclonal antibody	543	541
15	Open-label	541	524
16	Immunohistochemistry	525	521
17	Pertuzumab	522	519
18	Lapatinib	520	483
19	Docetaxel	483	474
20	Metastatic breast cancer	474	446

**Table 8:** Top 20 keywords in HER2+ research 2020–2024.

Rank	Keywords	Occurrences
1	Breast cancer	1,032
2	Trastuzumab	685
3	Chemotherapy	483
4	Survival	484
5	Her2	466
6	Therapy	428
7	Pertuzumab	315
8	Open-label	296
9	Expression	375
10	Multicenter	250
11	Women	238
12	Efficacy	209
13	Resistance	211
14	Lapatinib	183
15	Docetaxel	173
16	Combination	166
17	Metastatic breast cancer	187
18	Safety	160
19	Receptor	167
20	Pathological complete response	142

### Globally approved therapies for HER2+ BC

**Table 9** shows a summary of targeted therapies and chemotherapy options used as interventions in HER2+ BC. Trastuzumab, the pioneering monoclonal antibody, was the first FDA-approved targeted therapy in 1998 for HER2+ BC, followed by lapatinib, pertuzumab, and trastuzumab emtansine. The table shows their associated clinical trials and FDA approval numbers. The drug pyrotinib is not approved by the FDA but is approved and used in China for the treatment of HER2+ metastatic BC. Margetuximab is a recently approved (2020) monoclonal antibody indicated for patients with metastatic HER2+ BC. Eribulin is a chemotherapeutic agent that, when combined with trastuzumab, can be used to treat HER2+ advanced BC.

**Table 9:** Table showing global approved anti-HER2+ therapies.

	Generic drug name	Brand drug name	Drug Class	Clinical Study	Approval year	Publication DOI
	Trastuzumab	Herceptin	Monoclonal Antibody	HERA trial (NCT00045032)	1998	doi:10.1056/NEJMoa052306
	Lapatinib	Tykerb	Tyrosine Kinase Inhibitor	EGF100151 trial (NCT00078572)	2007	doi:10.1056/NEJMoa064320
	Pertuzumab	Perjeta	Monoclonal Antibody	CLEOPATRA trial (NCT00567190)	2012	doi:10.1056/NEJMoa1113216
**Targeted therapies**	Ado-Trastuzumab emtansine(T-DM1)	Kadcyla	Antibody-Drug Conjugate	EMILIA trial (TDM4370g) (NCT00829166)	2013	doi:10.1056/NEJMoa1209124
	Neratinib	Nerlynx	Tyrosine Kinase Inhibitor	ExteNET trial (NCT00878709)	2017	doi:10.1016/S1470-2045(15)00551-3
	Pyrotinib	Pyrotinib Mesylate	Tyrosine Kinase Inhibitor	PHOEBE trial (NCT03080805)	2018	doi:10.1016/S1470-2045(20)30702-6
	Trastuzumab deruxtecan(T-DXdb)	Enhertu	Antibody-Drug Conjugate	DESTINY-Breast01 trial (NCT03248492)	2019	doi:10.1056/NEJMoa1914510
	Tucatinib	Tukysa	Tyrosine Kinase Inhibitor	HER2CLIMB trial (NCT02614794)	2020	doi: 10.1056/NEJMoa1914609
	Margetuximab	Margenza	Monoclonal Antibody	SOPHIA trial (NCT02492711)	2020	doi:10.1200/JCO.21.02937
						
	Doxorubicin	Adriamycin	Anthracycline chemotherapy agent	NCT00005970	1974	doi:10.1200/JCO.2011.36.7045
**Chemotherapeutic agents**	Paclitaxel	Taxol	Taxane chemotherapy agent	NCT00542451	1992	doi:10.1056/NEJMoa1406281
	Docetaxel	Taxotere	Taxane chemotherapy agent	NCT00003773	1996	doi:10.1200/JCO.2002.07.058
	Capecitabine	Xeloda	Antimetabolite chemotherapy agent	NCT00174893	1998	doi:10.1200/JCO.2006.09.6826
	Eribulin mesylate	Halaven	Microtubule inhibitor chemotherapy agent	EMBRACE trial (NCT00388726)	2010	doi: 10.1016/S0140-6736(11)60070-6

## Discussion

The HER2 gene was first discovered in mice in 1984; however, it was not until 1987 that Slamon et al. discovered the link between HER2 and BC (12, 13). Initially, HER2+ BC was associated with aggressive disease and poor outcomes; however, the discovery of trastuzumab and improvements in precision oncology have led to dramatic prognostic improvement (14). To our knowledge, this study is the first bibliometric analysis of HER2+ BC. Analyzing the most cited and influential articles in HER2+ BC outlines the evolution of HER2+ BC and helps visualize emerging research trends, serving as a guide for clinicians on the current state and future direction of HER2 research.

The most cited paper on HER2+ BC is Slamon et al.’s 1987 paper (13), which established the correlation between HER2 and human BC. This paper found that 30% of breast tumors demonstrate amplification of the HER2 oncogene to greater than 20-fold even when other prognostic factors were controlled (13). Ultimately, the data from this study helped determine the impact of HER2 in the pathogenesis of BC. The next most cited papers focus on biological mechanisms aiming to ascertain the association between HER2 overexpression and prognosis. A 1989 study by J. Baselga et al. shed light on the role of Trastuzumab in enhancing the anti-tumor activity of paclitaxel and doxorubicin against HER2 amplified human BC xenografts (14). This publication precipitated a shift in focus from the efficacy of screening HER2+ BC towards exploiting HER2 as a therapeutic target. The importance of anti-HER2 therapies, both as single agents and in combination with other therapies, is demonstrated in this analysis with consistently high publication numbers. The current standard of care for adjuvant treatment of HER2+ BC, is dual anti-HER2 monoclonal antibodies (trastuzumab and pertuzumab) with docetaxel. This regimen was established largely on the basis of the promising results of the CLEOPTATRA trial, and a recent increase in citations highlights the magnitude of this trial’s impact on HER2+ BC (15). The similarly cited HERA trial reported no benefit in 2 years of trastuzumab treatment over 1 year, leading to guideline changes (16). This level of citation again reflects the impact this research has had on the landscape of HER2+ BC.

A total of 101 countries contributed to papers on the topic of HER2+ BC. The USA was the highest contributor, a somewhat expected finding given the HER2 gene and the association of HER2 positivity with BC and trastuzumab were all discovered there. Other factors such as the availability of resources and funding likely facilitated this high ranking. The top affiliations were the University of Texas system, the University of California, Los Angeles (UCL), Genentech, Memorial Sloan Kettering Cancer Center and Harvard University. These institutions have a long-standing interest in HER2+ BC research. Dennis Slamon, the founder of trastuzumab, is the chief of the division of Hematology-Oncology at UCLA, and Genentech is the company that developed trastuzumab.

“Metastatic breast cancer” is among the most commonly used keywords in the last 5 years, as despite overall treatment advances, the prognosis of metastatic BC remains poor. This is particularly relevant to HER2+ BC given the reduced efficacy of targeted HER2 in the metastatic setting. Development of resistance to anti-HER2 therapies has thus far posed an insurmountable therapeutic challenge, particularly in the context of advanced disease. However, the development of novel treatments including immunotherapy, cell-cycle inhibitors, and ADCs has improved outcomes of metastatic disease. ADCs have had particularly promising results in metastatic disease, and this is reflected by their high article publication and citation numbers of late. ADCs, such as trastuzumab deruxtecan (T-DXd) and trastuzumab emtansine (TDM-1), consist of a cell surface protein antigen, a cytotoxic agent, and a linker that combines them (17). The DESTINY-Breast03 trial, a multicenter randomized control trial (RCT), found that median progression-free survival with T-DXd was 28.8 months (95% CI 22.4–37.9) compared to 6.8 months (95% CI 5.6–8.2) in those treated with trastuzumab emtansine [hazard ratio 0.33 (95% CI 0.26–0.43), *p* < 0.0001] (18). TDXt is also currently being explored in the neoadjuvant setting for primary disease (19). The interest in research focused on overcoming resistance is clear in this study, with “resistance” identified as a top key term for the last 5 years. The centrality of Goel et al.’s article, “Overcoming therapeutic resistance in HER2-positive breast cancers with CD4/6 inhibitors”, in citation analysis affirms this focus (20). Several mechanisms of treatment resistance are recognized including activation of the PI3K/Akt/mTOR signaling pathway in response to prolonged treatment course, which may cause resistance through expression of mutated PTEN or PIK3CA genes (21). Detailed understanding of this pathway, gained through scientific exploration, has led to the FDA approval of capisertib, an Akt inhibitor (22). This exemplifies the importance of cohesive international efforts to elucidate the interaction between signaling pathways, tumor proliferation, and treatment response at a molecular level to identify druggable targets that improve patient outcomes. Other important mechanisms of resistance backed by numerous publications include Src mutations, MET mutations, and HER2 activating mutations (21).

Anti-HER2 therapy in the neoadjuvant setting is another focus of continually evolving HER2+ BC research. Dual trastuzumab and pertuzumab in combination with chemotherapy is prescribed both to downstage larger HER2+ primary tumors and to assess tumor response, guiding subsequent adjuvant therapies (23). The results of several ongoing trials assessing the efficacy of other therapeutic agents in achieving a pathological complete response and improving survival outcomes such as tyrosine kinase inhibitors, immunotherapy, and ADCs are eagerly awaited (19, 24, 25).

There are several limitations of this study. WoSCC was the only database that was used to search for manuscripts. While WoSCC has the broadest collection of literature and is one of the most widely used databases, it is possible that some articles have been omitted. Secondly, the level of evidence for each study was not evaluated as the present study aims to delineate the landscape of HER2+ BC research since its discovery in 1987 rather than provide a review of the literature itself. Lastly, the confounding factor of time since publication was not comprehensively analyzed, and thus, the citation numbers may be elevated in older publications as a result of having more time for citation rather than a true predominance of interest. An example is the omission of publications on HER2 vaccines in the treatment of BC. In 2022, NCT00436254 established the efficacy and safety of a plasmid DNA vaccine encoding the ERBB2 intracellular domain in late-stage HER2+ BC (26). NCT00791037 observed that T-cell infusion post HER2 DNA vaccine improved survival outcomes in patients with advanced disease (27). While failing to be recognized by the lists compiled in this study, this cutting-edge area of research may be among the most impactful in future management of HER2+ BC.

## Conclusion

The present study illustrates the evolution of HER2 since the discovery of its link with BC. The discovery of anti-HER2 therapies and subsequent improvements in patient outcomes highlights the importance of both clinical and lab-based research in BC. The US and US-based institutions have continued to publish the most impactful articles on HER2+ BC. Emerging trends in HER2+ BC research are treatment of metastatic HER2+ BC, overcoming therapy resistance, and targeting HER2 in the neoadjuvant setting.

## Author contributions

SA-T: Conceptualization, Formal analysis, Methodology, Project administration, Writing – original draft, Writing – review & editing. GD: Conceptualization, Methodology, Project administration, Supervision, Writing – review & editing. GD: Resources, Software, Writing – review & editing. CK: Conceptualization, Writing – review & editing. LC: Formal analysis, Writing – review & editing. JM: Formal analysis, Writing – review & editing. MA: Writing – review & editing. SN: Writing – review & editing. CP: Conceptualization, Supervision, Writing – review & editing. AH: Supervision, Writing – review & editing.
